# Identifying Profiles of Digital Literacy Among Community-Dwelling Korean Older Adults: Latent Profile Analysis

**DOI:** 10.2196/57122

**Published:** 2025-02-19

**Authors:** Jiyoung Shin, Hun Kang, Seongmi Choi, Sang Hui Chu, JiYeon Choi

**Affiliations:** 1 Mo-Im Kim Nursing Research Institute, College of Nursing, Yonsei University Seoul Republic of Korea; 2 Health Insurance Research Institute, National Health Insurance Service Gangwon Republic of Korea; 3 Department of Social and Behavioral Sciences Yale University School of Public Health New Haven, CT United States; 4 Research Institute of Nursing Science, College of Nursing, Daegu Catholic University Daegu Republic of Korea; 5 Institute for Innovation in Digital Healthcare, Yonsei University Seoul Republic of Korea

**Keywords:** digital literacy, digital divide, older adults, South Korea, latent profile analysis

## Abstract

**Background:**

The digital divide is apparent not only between older and younger generations but also within the older adult population itself. Identifying digital literacy profiles among older adults is crucial for developing targeted strategies to narrow this divide.

**Objective:**

This study aimed to identify profiles of digital literacy among community-dwelling older adults and to examine factors associated with these profiles.

**Methods:**

Data were collected from community-dwelling older adults in South Korea through a nationwide cross-sectional survey that assessed digital literacy and related factors. Digital literacy was evaluated across 3 domains: information and communication (9 items), content creation and management (4 items), and safety and security (9 items). Latent profile analysis was used to identify profiles of digital literacy among community-dwelling older adults, and multinomial logistic regression was used to identify predictors of profile membership.

**Results:**

A total of 1016 older adults completed structured questionnaires (average age 68, SD 6.5 years; 486/1016, 47.8% men). Three digital literacy profiles were identified (*P*<.001): “low level” (346/1016, 34.1%), “middle level” (374/1016, 36.8%), and “high level” (296/1016, 29.1%). With the “middle-level” digital literacy group as the reference group, older adult participants (odds ratio [OR] 1.11, 95% CI 1.07-1.15) with less than a middle school education (vs with a college degree or higher; OR 7.22, 95% CI 2.31-22.54), who needed help with one of the 10 instrumental daily activities (vs ≥2 activities; OR 3.06, 95% CI 1.11-8.40) and who did not engage in in-person social activities (OR 1.81, 95% CI 1.07-3.07), were more likely to be in the “low-level” group. Women were less likely to be in the “high-level” digital literacy group than men (OR 0.45, 95% CI 0.25-0.80). Participants with less than a college education were also less likely to be in the “high-level” group, with those having less than a middle school education showing the lowest OR (OR 0.17, 95% CI 0.07-0.41). Those who had never worked (OR 0.23, 95% CI 0.06-0.90) and those not engaging in regular physical exercise (OR 0.58, 95% CI 0.40-0.84) were also less likely to be in the “high-level” digital literacy group. Participants with greater social support were more likely to be in the “high-level” digital literacy group (OR 1.70, 95% CI 1.22-2.37).

**Conclusions:**

These findings underscore the characteristics linked to lower digital literacy and suggest a tailored approach to meet the needs of diverse groups of older adults in a digitalizing society. To promote digital literacy among older adults, potential strategies include improving access to and guidance for using digital devices, specifically designed for this demographic, as well as promoting social support and encouraging participation in social activities.

## Introduction

### Background

In a digitalizing world, new technology-supported lifestyles hold the potential to foster healthy and independent aging among older adults. These individuals can manage their health and medical conditions through mobile devices [1] or benefit from telecare and access to online health information [2]. In addition, smart homes powered by the Internet of Things can support the daily activities of older adults [3]. Digital devices also enable older adults to maintain social connections and participate in social activities, even when traditional face-to-face interactions are not possible [4]. Therefore, it is crucial to encourage older adults to embrace digital technology to enhance self-care, functionality, and social connection [5].

Nevertheless, the digital divide remains a substantial challenge in our increasingly digital world [6]. Research consistently shows a gap in digital engagement between older adults and younger generations [7,8]. Furthermore, there is a considerable variation in digital proficiency among older adults themselves. For instance, individuals aged ≥75 years generally demonstrated lower levels of digital use and literacy compared to those aged <75 years [9]. In addition, factors such as race and ethnicity and socioeconomic status significantly influence internet use among older adults [10]. To effectively support older adults in adapting to and participating in the digital society, it is essential to recognize and understand the diversity within this demographic.

To address this knowledge gap, it is necessary to understand the varied characteristics of digital literacy within the population and to identify the factors associated with it. Previous research on digital literacy in older adults has primarily focused on their abilities to search for information and communicate online [11-13]. However, the definition of digital literacy is evolving [14]. It is now described as “the ability to access, manage, understand, integrate, communicate, evaluate, and create information safely and appropriately through digital technologies” [15]. In response, the European Commission has introduced a detailed self-assessment grid within the Digital Competence (DigComp) framework. This framework covers a broader range of skills, including content creation and management as well as safety and security, in addition to information search and online communication [11,16]. Therefore, conducting a comprehensive assessment of digital literacy among older adults is both relevant and timely.

Previous studies have primarily focused on identifying factors associated with digital literacy levels among older adults, treating this population as uniform. These studies revealed that not only sociodemographic factors—such as gender [17], age [18,19], living arrangement [8], education level [18], and region [8,19]—but also health-related factors, including cognitive functioning [20], physical limitations [21], and depressive symptoms [22], as well as social factors, such as social support [23] and social participation [24], are linked to digital literacy. However, this method may overlook the varied expressions of digital literacy within the older adult population. A person-centered approach that recognizes this diversity is essential for developing comprehensive education and training programs aimed at enhancing digital literacy among older adults [25].

### Objective

Therefore, this study, grounded in the most recent definition of digital literacy, aims to achieve three objectives: (1) identify the profiles of digital literacy among community-dwelling older adults in South Korea, (2) understand the characteristics of digital literacy profiles, and (3) determine the predictive factors that distinguish these profiles.

## Methods

### Study Design and Setting

This cross-sectional study aimed to assess the digital literacy levels and associated factors among community-dwelling older adults in South Korea. Data were collected through a nationwide survey conducted from October to November 2022 [26]. The survey used proportional stratified sampling on the basis of region, sex, and age groups, reflecting the population distribution documented in South Korea as of June 2022. The inclusion criteria were (1) being aged ≥60 years, (2) achieving a minimum score of 22 on the Korean version of the Mini-Mental State Examination (second edition), and (3) being proficient in the Korean language. In total, 58 trained interviewers visited the homes of potential participants identified through the sampling process. They provided comprehensive information about the study and obtained informed consent from those who were eligible and willing to participate. A total of 1016 older adults completed structured questionnaires designed by the research team to collect data on digital literacy, sociodemographic characteristics, health status, health behaviors, social activities, and social support. The survey was conducted via one-on-one interviews using tablet PCs, with an average completion time of 26.8 minutes per participant. Interviews were conducted in person by trained interviewers with extensive experience in interviewing older adults. Errors or inaccurate responses identified during the review or verification process of completed surveys were corrected or supplemented by interviewers through follow-up phone inquiries.

### Ethical Considerations

In compliance with the Helsinki Declaration, this study received approval from the Institutional Review Board of Yonsei University (4-2022-0396). All participants provided written informed consent before their involvement in the study and received gift vouchers worth 10,000 Korean won (US $6.90) upon completing the survey. The collected data were made accessible only to the research team, and the respondents’ personal information was deidentified before analysis.

### Measures

#### Digital Literacy

In this study, we evaluated the components of digital literacy in older adults using the Everyday Digital Literacy Questionnaire (EDLQ), which consists of 22 items developed from our earlier research based on the European Commission’s DigComp framework [26]. The EDLQ has shown satisfactory validity and reliability, and it is organized into 3 domains: information and communication (9 items), content creation and management (4 items), and safety and security (9 items) [26]. Responses to each item were recorded on a Likert scale from 1 (strongly disagree) to 5 (strongly agree), with higher aggregate scores indicating greater digital literacy. In our sample, the Cronbach α value was 0.98, confirming high internal consistency [26].

#### Predictor Variables of Digital Literacy

##### Sociodemographic Factors

On the basis of previous studies [8,17-19], several sociodemographic factors were identified as influencing the digital literacy of older adults. These factors included age, sex, educational level, living arrangements, region, and economic activities. Age was treated as a continuous variable, measured in years. Sex was categorized as a binary variable, with male coded as 1 and female coded as 0. Educational levels were divided into 4 categories: below middle school graduation (coded as 0), below high school graduation (coded as 1), below college graduation (coded as 2), and college graduation or higher (coded as 3). Living arrangements were classified into 3 categories: living alone (coded as 0), living with a spouse (coded as 1), and residing in a multigenerational household (coded as 2). Region was split into 2 categories: metropolitan areas (coded as 1), which included Seoul, Incheon, and Gyeonggi province, and nonmetropolitan areas (coded as 0), encompassing cities and provinces outside the metropolitan area. Economic activities were categorized into 3 groups: currently engaged in income-generating activities (coded as 2), previously employed but not currently (coded as 1), and never having worked (coded as 0).

##### Health Factors

On the basis of previous studies [20-22], variables related to health status and behavior were selected as factors influencing the digital literacy of older adults.

For health status, participants provided information on their cognitive, physical, and psychological health. The cognitive health of the participants was assessed using the Korean version of the Mini-Mental State Examination (second edition) [27]. Following the most widely used cutoff point for screening the extent of cognitive impairment [28], we recoded the scores into a binary variable: 24 or higher (coded as 1) and below 24 (coded as 0).

The physical health of the participants was assessed, covering disabilities, chronic diseases, and physical function. Disability status was determined as a binary variable, indicating whether participants had received an official medical diagnosis from specialty physicians: yes (coded as 1) or no (coded as 0). In South Korea, the Act on Welfare of Persons with Disabilities defines a “person with a disability” as someone whose daily life or social activity is significantly impaired by a physical or mental disability over a long period. This act recognizes 15 medical disability categories, including disabilities of the extremities; vision disability; hearing disability; speech and language disability; disability due to facial deformity; intellectual disability; and disabilities resulting from brain injury, renal failure, heart problems, liver disease, respiratory issues, ostomy, epilepsy, autism, and mental disorders [29]. Chronic diseases were categorized into 3 groups based on the number of chronic conditions that lasted over 3 months as diagnosed by a physician: none (coded as 0); 1 (coded as 1); and ≥2 (coded as 2). Physical function was evaluated from 2 perspectives using the Korean-Activities of Daily Living (K-ADL) and the Korean-Instrumental Activities of Daily Living (K-IADL). The K-ADL assesses the level of assistance needed in 7 basic activities of daily living: dressing, washing face and hands, bathing, eating, transferring, toileting, and continence. The Cronbach α value for K-ADL at the time of its development was 0.94 [30], and in this study, it was 0.75. The K-IADL measures the level of assistance needed in 10 instrumental activities of daily living, including decorating, housework, meal preparation, laundry, short-distance travel, using transportation, shopping, handling money, using the telephone, and managing medication. The Cronbach α value for K-IADL at the time of its development was 0.94 [31], and in this study, it was 0.78. We recoded the number of items requiring assistance in K-ADL and K-IADL into 3 categories for each: none (coded as 0); 1 (coded as 1), and ≥2 (coded as 2).

The psychological health of the participants was assessed through health-related quality of life and depressive symptoms. Health-related quality of life was evaluated using the Short-Form Health Survey, which examines 8 domains: physical functioning, role-physical, bodily pain, general health, vitality, social functioning, role-emotional, and mental health. For our study, we used version 2 of the 12-item Short-Form Health Survey, an abbreviated version of the 36-item Short-Form Health Survey [32]. Scores were derived using published algorithms that set the mean score of the US general population at 50 and the SD at 10, which were used to calculate the physical component summary and mental component summary scores [32,33]. Scores ranged from 0 to 100, with higher scores indicating a better quality of life. Depressive symptoms were assessed using the integrated Korean version of the Center for Epidemiological Studies-Depression (CES-D) [34]. Participants rated the frequency of their feelings and behaviors over the past week for a total of 20 items, ranging from “rarely or never (<1 day)” (coded as 0) to “most or all of the time (5-7 days)” (coded as 3). After reverse coding the 4 positively worded items, we calculated the total score from the 20 responses. Using the widely accepted threshold for identifying individuals at risk of clinical depression (ie, ≥16) [35], we categorized participants into 2 groups: those at risk for clinical depression and those not at risk. The Cronbach α values for the original CES-D scale and its Korean version were 0.85 and 0.91, respectively [34,36]. In our study sample, the Cronbach α was determined to be 0.89.

For health behaviors, participants provided information on smoking, alcohol consumption, and physical activity. Participants were categorized into 3 groups based on their smoking habits: nonsmokers (coded as 0), ex-smokers (coded as 1), and current smokers (coded as 2). Regarding alcohol consumption, participants indicated whether they had consumed alcohol at least once in the past year (coded as 1) or not (coded as 0). Physical activity was assessed by determining whether participants engaged in daily physical exercise for 10 minutes or more on a regular basis (coded as 1) or not (coded as 0).

##### Social Factors

On the basis of prior studies [23,24], variables, such as participation in in-person and digital social activities, along with social support, were included as social factors influencing digital literacy among older adults.

To understand participants’ in-person social activities, we inquired about their participation over the past year in various activities, including religious or social gatherings; leisure, culture, or sports activities; alumni meetings; volunteer work; political, civic, interest group activities; or other activities [37]. Participants indicated the annual frequency of their involvement in each category of social activities using a scale ranging from “several times a week” (coded as 1) to “not at all” (coded as 7). For our analysis, we grouped these responses into 2 categories: nonparticipation (coded as 0), which includes only the responses indicating “not at all” for all social activity categories, and participation (coded as 1), which includes all other responses. We also evaluated participants’ digital social activities by examining their use of digital devices such as desktops and laptops, mobile phones, tablets, e-books, and wearable devices. Participants were asked whether they had used each of the 5 types of digital devices and to specify the duration of use in months. The longest duration reported by each participant was recorded as a continuous variable to represent their overall digital device use. Responses from 10.5% (107/1016) of participants who had no experience using any digital device were recorded as 0 months.

We used the Multidimensional Scale of Perceived Social Support (MSPSS) to assess perceived social support from family, friends, and significant others. The original scale featured a 7-point Likert scale for its 12 items, ranging from “very strongly disagree” (coded as 1) to “very strongly agree” (coded as 7) [38]. For our study, we used the Korean translated version of the MSPSS, which modified the scale to range from “strongly disagree” (coded as 1) to “strongly agree” (coded as 5) [39]. Higher average scores indicate stronger perceived social support. The Cronbach α values were 0.88 for the original version, 0.89 for the Korean translated version, and 0.94 for our sample.

#### Data Analysis

We chose latent profile analysis (LPA) as the analytical method to identify profiles of digital literacy among older adults living in the community. LPA is a person-centered clustering approach that classifies individuals into unobserved latent subgroups on the basis of similar profiles of observed continuous variables [40]. Our sample size met the minimum recommended criterion (n≥500) for accurate identification using LPA [40,41]. We used 22 items measuring digital literacy across 3 domains as indicator variables for this analysis.

In the initial step of LPA, we selected the model by using information criteria, classification quality indexes, and relative fit indexes. We calculated the Bayesian information criterion, Akaike information criterion, and sample size–adjusted Bayesian information criterion as our information criteria, with lower values indicating a better-fitting model [42]. To assess the quality of model classification, we analyzed the entropy value, targeting a criterion of ≥0.8 [43]. To determine if significant differences in model fit existed between models with K-1 and K profiles, we evaluated the *P* values (*P*<.05) using the Lo-Mendell-Rubin likelihood ratio test, adjusted Lo-Mendell-Rubin likelihood ratio test, and bootstrapped likelihood ratio test [44]. Subsequently, we chose the most suitable number of profiles on the basis of the goodness-of-fit results and their interpretability. Finally, we incorporated predictor variables that affect digital literacy as auxiliary variables to reduce classification errors among profiles [45]. The LPA process was carried out using Mplus (version 8.8; Muthén & Muthén).

To compare the characteristics of the profiles identified through LPA and determine if there were statistically significant differences, we conducted Kruskal-Wallis *H* tests and chi-square tests. In addition, to identify predictor variables that differentiate each profile, we estimated the odds ratio (OR) and their corresponding 95% CIs for the probability of belonging to a specific profile membership using a multinomial logistic regression model. The analysis for intergroup comparisons was performed using SPSS Statistics (version 26; IBM Corp).

## Results

We analyzed data from 1016 community-dwelling older adults in South Korea who completed the survey. The participants’ average age was 68.0 (SD 6.5) years, and 47.83% (486/1016) of participants were men.

### Identification of Digital Literacy Profiles

#### Model Selection: 3-Class Model

Table 1 presents the available model selection scenario for our sample. The Bayesian information criterion, Akaike information criterion, and sample size–adjusted Bayesian information criterion values decreased as the number of profiles increased, with the rate of decrease diminishing after the 3-profile point. The entropy values exceeded 0.8 in all simulations, indicating satisfactory classification quality. Relative fit indexes demonstrated significant fit only in the 2-profile, 3-profile, and 4-profile scenarios across all 3 indexes (all *P*=.002 or <.001). Overall, the model evaluation results suggested that both the 3-profile and 4-profile models were statistically appropriate. However, given the theoretical interpretability of the profile characteristics, the 3-profile model was deemed the most suitable for our sample.

**Table 1:** Summary of model fit indexes for latent profile analysis.

Fit indexes	1 profile	2 profile	3 profile	4 profile	5 profile	6 profile
AIC^a^	75,974.05	61,809.16	*57,344.01* ^b^	55,771.28	54,597.69	53,771.08
BIC^c^	76,190.69	62,139.05	*57,787.14*	56,327.65	55,267.30	54,553.94
SSABIC^d^	76,050.94	61,926.25	*57,501.29*	55,968.75	54,835.353	54,048.94
Entropy	—^e^	0.982	*0.968*	0.954	0.958	0.954
LMR-LRT^f^, *P* value	—	<.001	*<.001*	.002	.18	.049
Adjusted LMR-LRT, *P* value	—	<.001	*<.001*	.002	.18	.051
BLRT^g^, *P* value	—	<.001	*<.001*	<.001	<.001	<.001

^a^AIC: Akaike information criterion.

^b^Selected class solution values are italicized.

^c^BIC: Bayesian information criterion.

^d^SSABIC: sample size–adjusted Bayesian information criterion.

^e^Not applicable.

^f^LMR-LRT: Lo-Mendell-Rubin likelihood ratio test.

^g^BLRT: bootstrapped likelihood ratio test.

#### Describing Digital Literacy Profiles

As shown in Table 2, the indicators of digital literacy revealed statistically significant differences across the 3 profiles (all *P* values <.001). Profile 1, which comprises the second-largest group (346/1016, 34.1%), demonstrated the lowest digital literacy levels, with mean scores <2 (out of 5) for each item. Within profile 1, the items “social networking” (mean 1.78), “find information on the Internet” (mean 1.77), and “evaluate internet information” (mean 1.71) in the information and communication domain showed relatively higher self-reported proficiency. Conversely, “convert document formats” (mean 1.12), “edit and share content” (mean 1.13), and “troubleshoot device issues” (mean 1.13) in the content creation and management domain, along with “block spam/phishing” (mean 1.13) in the security and safety domain, scored relatively lower.

Profile 2, the largest group, comprising 36.8% (374/1016) of the sample, exhibited median scores across all items when compared to the other 2 profiles. The mean scores for each item varied significantly, ranging from 1.86 to 3.56. Notably, within the information and communication domain, the items “find information on the Internet” (mean 3.56) and “social networking” (mean 3.54) achieved higher mean scores, both exceeding a mean of 3.5. Conversely, in the content creation and management domain, items such as “edit and share content” (mean 1.86), “convert document formats” (mean 1.89), “troubleshoot device issues” (mean 1.99), and “create documents” (mean 2.10) recorded lower mean scores.

The smallest group, profile 3 (296/1016, 29.1%), comprised participants with the highest relative level of digital literacy. Although the difference was not substantial compared to profile 2, there was a notable variation in the mean scores among the items within this group. All items in the domains of information and communication and safety and security scored a high mean, around 4 (ranging from 3.83 to 4.33), with the exception of “seeking technical help,” which scored a mean of 3.63. However, similar to the other 2 profiles, items in the content creation and management domain exhibited the lowest relative scores, ranging from 3.10 to 3.56.

**Figure 1:**
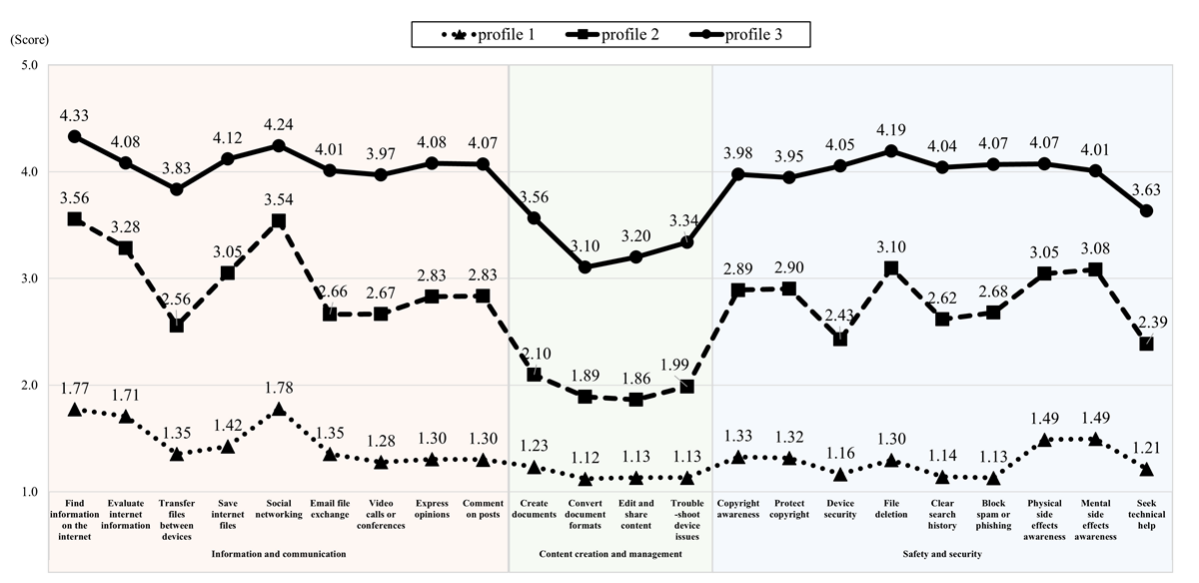
The distribution of digital literacy mean scores among 3 latent profiles.

Figure 1 illustrates the distribution of mean scores for digital literacy indicators across 3 latent profiles. The x-axis represents the measurement items from the 3 domains, while the y-axis shows response scores ranging from 1 to 5 points. On the basis of the distribution characteristics of the 22-item EDLQ scores that measure digital literacy, the profiles were labeled as follows: profile 1 as “low level” (346/1016, 34.1%), profile 2 as “middle level” (374/1016, 36.8%), and profile 3 as “high level” (296/1016, 29.1%).

**Table 2:** Mean scores^a^ of digital literacy in each latent profile.

Variables			Profile 1: low level (n=346), mean (SD)		Profile 2: middle level (n=374), mean (SD)		Profile 3: high level (n=296), mean (SD)		Kruskal-Wallis *H* test		*P* value
**Information and communication**											
	Find information on the Internet	1.77 (0.96)		3.56 (0.81)		4.33 (0.57)		620.78		<.001	
	Evaluate internet information	1.71 (0.93)		3.28 (0.89)		4.08 (0.74)		552.68		<.001	
	Transfer files between devices	1.35 (0.61)		2.56 (1.06)		3.83 (0.85)		575.33		<.001	
	Save internet files	1.42 (0.69)		3.05 (0.98)		4.12 (0.72)		648.31		<.001	
	Social networking	1.78 (1.07)		3.54 (0.90)		4.24 (0.63)		539.37		<.001	
	Email file exchange	1.35 (0.69)		2.66 (1.18)		4.01 (0.90)		559.30		<.001	
	Video calls or conferences	1.28 (0.59)		2.67 (1.07)		3.97 (0.84)		617.49		<.001	
	Express opinions	1.30 (0.59)		2.83 (1.00)		4.08 (0.75)		663.83		<.001	
	Comment on posts	1.30 (0.57)		2.83 (1.04)		4.07 (0.78)		656.01		<.001	
**Content creation and management**											
	Create documents	1.23 (0.57)		2.10 (0.94)		3.56 (1.03)		545.76		<.001	
	Convert document formats	1.12 (0.34)		1.89 (0.82)		3.10 (1.06)		520.24		<.001	
	Edit and share content	1.13 (0.38)		1.86 (0.81)		3.20 (1.08)		523.36		<.001	
	Troubleshoot device issues	1.13 (0.38)		1.99 (0.81)		3.34 (0.86)		620.99		<.001	
**Safety and security**											
	Copyright awareness	1.33 (0.73)		2.89 (1.06)		3.98 (0.82)		608.33		<.001	
	Protect copyright	1.32 (0.69)		2.90 (1.00)		3.95 (0.78)		631.26		<.001	
	Device security	1.16 (0.41)		2.43 (0.92)		4.05 (0.73)		733.66		<.001	
	File deletion	1.30 (0.70)		3.10 (0.99)		4.19 (0.70)		681.36		<.001	
	Clear search history	1.14 (0.39)		2.62 (0.93)		4.04 (0.83)		732.09		<.001	
	Block spam or phishing	1.13 (0.39)		2.68 (0.95)		4.07 (0.80)		730.69		<.001	
	Physical side effects awareness	1.49 (0.90)		3.05 (1.05)		4.07 (0.73)		569.83		<.001	
	Mental side effects awareness	1.49 (0.93)		3.08 (1.05)		4.01 (0.71)		548.17		<.001	
	Seek technical help	1.21 (0.56)		2.39 (1.03)		3.63 (0.80)		598.24		<.001	

^a^Item scores range from 1 to 5.

### Comparison of Participant Characteristics Among Digital Literacy Profiles

Table 3 provides an overview of the participant characteristics and the differences between the groups in the adopted 3-profile model. The average age of the participants was 68 (SD 6.5) years. Among the total number of participants (N=1016), the majority were female (n=530, 52.2%), had completed at least high school (n=551, 54.2%), lived with a spouse (n=524, 51.6%), resided in nonmetropolitan areas (n=547, 53.8%), and were engaged in income-generating activities (n=622, 61.2%). Most participants had no cognitive impairment (n=969, 95.4%), had been diagnosed with one or more chronic diseases (n=704, 69.3%), and had no diagnosed disabilities (n=967, 95.2%). They were capable of performing independent activities of daily living (n=952, 93.7%) and instrumental activities of daily living (n=839, 82.6%). Regarding health-related quality of life, the mean score for the physical aspect was 50.1 (SD 7.3; out of 100), and for the mental aspect, it was 49.4 (SD 8.4; out of 100). Most participants were not at risk of clinical depression (n=750, 73.8%), engaged in regular physical exercise for more than 10 minutes a day (n=594, 58.5%), had not consumed alcohol in the past year (n=516, 50.8%), and were nonsmokers (n=628, 61.8%). Most participants reported having participated in in-person social activities over the past year (n=853, 84%), and the average perceived social support score was 3.9 (SD 0.7; out of 5). Participants reported an average digital device use history of 82.9 (SD 64.9) months.

When comparing participant characteristics across the 3 profiles, statistically significant differences were observed in sociodemographic, health, and social factors on the basis of digital literacy levels, with the exceptions of region and ADL (both *P* values>.05). In the group with “high-level” digital literacy, the average age was the lowest at 64.7 (SD 4.6) years, and the majority (260/296, 87.8%) had attained at least a high school education. In addition, this group had a higher proportion of male individuals (190/296, 64.2%) and a greater participation in current income-generating activities (217/296, 73.3%) than the other 2 groups. Conversely, in the group with “low-level” digital literacy, a higher percentage of participants had education levels below high school (277/346, 80.1%) and a larger proportion lived alone (117/346, 33.8%), relative to the other 2 groups.

Distinct health patterns were observed among the profiles analyzed. In the “high-level” digital literacy group, a higher proportion of participants exhibited no cognitive impairment (294/296, 99.3%), no diagnosed chronic conditions (120/296, 40.5%), no disabilities (286/296, 96.3%), and no depressive symptoms (244/296, 82.4%) compared to the other 2 groups. This group also scored highest in health-related quality of life, both physically and mentally (physical component summary: mean 53.2, SD 5.0; mental component summary: mean 51.4, SD 7.9), indicating a quality of life that surpasses the average of the reference group. In contrast, the “low-level” digital literacy group displayed poorer outcomes across these health indicators. Although there was no significant difference in ADL among the 3 groups, IADL differed significantly. Notably, the “low-level” digital literacy group had a higher proportion of participants reporting dependency in one or more IADL items (96/346, 27.8%) compared to the other groups. In terms of health behaviors, the “high-level” digital literacy group had a relatively higher proportion of participants who engaged in regular exercise for more than 10 minutes (211/296, 71.3%), consumed alcohol (183/296, 61.8%), and were current smokers (48/296, 16.2%) than the other groups.

Regarding social factors, the “high-level” digital literacy group had the highest proportion of participants engaging in in-person social activities than the other 2 groups, at 92.2% (273/296). In addition, this group reported the longest history of using digital devices, averaging 128.0 (SD 74.8) months, and the highest mean score for perceived social support, at a mean of 4.1 (SD 0.7; out of 5).

**Table 3:** Participants’ characteristics and differences among the 3 latent profiles.

Variables		Total (N=1016)	Profile 1: low level (n=346)	Profile 2: middle level (n=374)	Profile 3: high level (n=296)	*P* value	
Age (y), mean (SD)		68.0 (6.5)	72.5 (7.0)	66.5 (4.8)	64.7 (4.6)	<.001	
**Sex, n (%)**							<.001
	Male	486 (47.8)	129 (37.3)	167 (44.7)	190 (64.2)		
	Female	530 (52.2)	217 (62.7)	207 (55.3)	106 (35.8)		
**Education level, n (%)**							<.001
	Below middle school	240 (23.6)	183 (52.9)	44 (11.8)	13 (4.4)		
	Below high school	225 (22.1)	94 (27.2)	108 (28.9)	23 (7.8)		
	Below college	451 (44.4)	63 (18.2)	202 (54)	186 (62.8)		
	College and above	100 (9.8)	6 (1.7)	20 (5.3)	74 (25)		
**Living arrangements, n (%)**							<.001
	Living alone	199 (19.6)	117 (33.8)	48 (12.8)	34 (11.5)		
	Living with a spouse	524 (51.6)	156 (45.1)	209 (55.9)	159 (53.7)		
	Multigenerational household	293 (28.8)	73 (21.1)	117 (31.3)	103 (34.8)		
**Region, n (%)**							.15
	Metropolitan area^a^	469 (46.2)	145 (41.9)	180 (48.1)	144 (48.6)		
	Nonmetropolitan area^b^	547 (53.8)	201 (58.1)	194 (51.9)	152 (51.4)		
**Engaged in economic activities, n (%)**							<.001
	Never	40 (3.9)	19 (5.5)	18 (4.8)	3 (1)		
	Used to	354 (34.8)	157 (45.4)	121 (32.4)	76 (25.7)		
	Yes	622 (61.2)	170 (49.1)	235 (62.8)	217 (73.3)		
**K-MMSE-2^c^, n (%)**							<.001
	≥24	969 (95.4)	309 (89.3)	366 (97.9)	294 (99.3)		
	22-23	47 (4.6)	37 (10.7)	8 (2.1)	2 (0.7)		
**Number of diagnosed chronic diseases, n (%)**							<.001
	0	312 (30.7)	60 (17.3)	132 (35.3)	120 (40.5)		
	1	301 (29.6)	90 (26.0)	113 (30.2)	98 (33.1)		
	≥2	403 (39.7)	196 (56.6)	129 (34.5)	78 (26.4)		
**Disability, n (%)**							0.006
	No	967 (95.2)	319 (92.2)	363 (97.1)	285 (96.3)		
	Yes	49 (4.8)	27 (7.8)	11 (2.9)	11 (3.7)		
**Number of dependent items of K-ADL^d^, n (%)**							.20
	0	952 (93.7)	320 (92.5)	352 (94.1)	280 (94.6)		
	1	37 (3.6)	11 (3.2)	15 (4)	11 (3.7)		
	≥2	27 (2.7)	15 (4.3)	7 (1.9)	5 (1.7)		
**Number of dependent items of K-IAD^e^, n (%)**							<.001
	0	839 (82.6)	250 (72.3)	332 (88.8)	257 (86.8)		
	1	68 (6.7)	38 (11)	16 (4.3)	14 (4.7)		
	≥2	109 (10.7)	58 (16.8)	26 (7)	25 (8.4)		
**HRQoL^f^ (scores), mean (SD)**							
	PCS^g^	50.1 (7.3)	46.4 (8.2)	50.9 (6.5)	53.2 (5.0)	<.001	
	MCS^h^	49.4 (8.4)	47.5 (8.8)	49.7 (8.0)	51.4 (7.9)	<.001	
**CES-D^i^ scale (scores), n (%)**							<.001
	1-15	750 (73.8)	219 (63.3)	287 (76.7)	244 (82.4)		
	≥16	266 (26.2)	127 (36.7)	87 (23.3)	52 (17.6)		
**Exercise, n (%)**							<.001
	No	422 (41.5)	168 (48.6)	169 (45.2)	85 (28.7)		
	Yes	594 (58.5)	178 (51.4)	205 (54.8)	211 (71.3)		
**Alcohol consumption, n (%)**							<.001
	No	516 (50.8)	215 (62.1)	188 (50.3)	113 (38.2)		
	Yes	500 (49.2)	131 (37.9)	186 (49.7)	183 (61.8)		
**Smoking, n (%)**							<.001
	Nonsmoker	628 (61.8)	247 (71.4)	228 (61.0)	153 (51.7)		
	Ex-smoker	260 (25.6)	70 (20.2)	95 (25.4)	95 (32.1)		
	Current smoker	128 (12.6)	29 (8.4)	51 (13.6)	48 (16.2)		
**In-person social activities, n (%)**							<.001
	No	163 (16)	96 (27.7)	44 (11.8)	23 (7.8)		
	Yes	853 (84)	250 (72.3)	330 (88.2)	273 (92.2)		
History of digital device use (months), mean (SD)		82.9 (64.9)	43.3 (42.4)	84.0 (47.9)	128.0 (74.8)	<.001	
MSPSS^j^ (scores), mean (SD)		3.9 (0.7)	3.6 (0.8)	3.9 (0.7)	4.1 (0.7)	<.001	

^a^Metropolitan area: Seoul, Incheon, and Gyeonggi province.

^b^Nonmetropolitan area: cities and provinces other than the metropolitan area.

^c^K-MMSE-2: Korean Mini-Mental State Examination, second edition.

^d^K-ADL: Korean Activities of Daily Living.

^e^K-IADL: Korean Instrumental Activities of Daily Living.

^f^HRQoL: health-related quality of life.

^g^PCS: physical component summary.

^h^MCS: mental component summary.

**^i^**CES-D: Center for Epidemiological Studies-Depression.

^j^MSPSS: Multidimensional Scale of Perceived Social Support.

### Predictor Variables for Identifying Digital Literacy Profiles

Table 4 represents the results of multinomial logistic regression for predictor variables of digital literacy profiles. The statistically significant predictors that differentiate each group are age, sex, educational level, income-generating activities, dependence on IADL, physical health-related quality of life, physical exercise, in-person social activities, history of digital device usage, and perceived social support.

Among these variables, participants with an educational level below middle school were over 7 times more likely to be categorized in the “low-level” digital literacy group than the “middle-level” group compared to those holding a college degree or higher (OR 7.223, 95% CI 2.314-22.541). In addition, participants who required assistance with 1 of 10 instrumental activities of daily living, such as housework, meal preparation, or laundry, were over 3 times more likely to fall into the “low-level” digital literacy group than those requiring assistance with 2 or more such activities (OR 3.055, 95% CI 1.111-8.399). Furthermore, participants who did not engage in in-person social activities were nearly twice as likely to be in the “low-level” digital literacy group compared to those who did participate (OR 1.810, 95% CI 1.068-3.065). Each 1-month increment in the history of digital device use was associated with a lower likelihood of being in the “low-level” than “middle-level” digital literacy group (OR 0.986, 95% CI 0.981-0.991).

**Table 4:** Multinomial logistic regression for predicting variables of digital literacy profiles.

Variables			“Low level” vs “middle level” (reference)					“High level” vs “middle level” (reference)		
			OR^a^ (95% CI)		*P* value		OR (95% CI)			*P* value
Age (y)			1.109 (1.066-1.153)		<.001		0.945 (0.902-0.991)			.02
**Sex (reference: male)**										
	Female	0.683 (0.367-1.271)		.23		0.449 (0.253-0.795)			.01	
**Education level (reference: college and above)**										
	Below middle school	7.223 (2.314-22.541)		.001		0.166 (0.068-0.405)			<.001	
	Below high school	2.465 (0.812-7.487)		.11		0.106 (0.050-0.223)			<.001	
	Below college	1.725 (0.568-5.237)		.34		0.332 (0.179-0.614)			<.001	
**Living arrangements (reference: multigenerational household)**										
	Living alone	1.582 (0.880-2.846)		.13		1.669 (0.892-3.125)			.11	
	Living with a spouse	0.977 (0.613-1.559)		.92		1.080 (0.712-1.638)			.72	
**Region (reference: metropolitan area^b^)**										
	Nonmetropolitan area^c^	0.797 (0.535-1.188)		.27		0.923 (0.627-1.360)			.69	
**Engaged in economic activities (reference: yes)**										
	Never	0.825 (0.298-2.284)		.71		0.234 (0.061-0.902)			.04	
	Used to	0.894 (0.574-1.392)		.62		1.043 (0.671-1.623)			.85	
**K-MMSE-2^d^ (reference: 22-23)**										
	≥24	0.703 (0.282-1.748)		.45		0.612 (0.117-3.207)			.56	
**Number of diagnosed chronic diseases (reference: ≥2)**										
	0	0.647 (0.388-1.077)		.09		0.949 (0.592-1.522)			.83	
	1	0.920 (0.584-1.449)		.72		1.255 (0.787-2.002)			.34	
**Disability (reference: yes)**										
	No	0.601 (0.236-1.534)		.29		1.020 (0.326-3.187)			.97	
**Number of dependent items of K-ADL^e^ (reference: ≥2)**										
	0	1.110 (0.292-4.212)		.88		0.558 (0.131-2.380)			.43	
	1	0.356 (0.065-1.957)		.24		0.886 (0.168-4.669)			.89	
**Number of dependent items of K-IADL^f^ (reference: ≥2)**										
	0	1.044 (0.494-2.208)		.91		0.501 (0.235-1.066)			.07	
	1	3.055 (1.111-8.399)		.03		0.513 (0.175-1.501)			.22	
**HRQoL^g^**										
	PCS^h^	0.973 (0.943-1.004)		.09		1.047 (1.010-1.085)			.01	
	MCS^i^	0.986 (0.956-1.017)		.36		1.010 (0.981-1.040)			.49	
**CES-D^j^ (reference: ≥16)**										
	1-15	1.162 (0.684-1.972)		.58		0.892 (0.516-1.544)			.68	
**Exercise (reference: yes)**										
	No	0.953 (0.645-1.410)		.81		0.578 (0.397-0.843)			.004	
**Alcohol consumption (reference: yes)**										
	No	0.751 (0.496-1.137)		.18		0.938 (0.629-1.399)			.76	
**Smoking (reference: current smoker)**										
	Nonsmoker	1.725 (0.821-3.623)		.15		1.803 (0.938-3.466)			.08	
	Ex-smoker	0.814 (0.408-1.621)		.56		1.082 (0.601-1.946)			.79	
**In-person social activities (reference: yes)**										
	No	1.810 (1.068-3.065)		.03		1.058 (0.563-1.987)			.86	
History of digital device use			0.986 (0.981-0.991)		<.001		1.009 (1.006-1.013)			<.001
MSPSS^k^			0.813 (0.597-1.109)		.19		1.700 (1.219-2.369)			.002

^a^OR: odds ratio.

^b^Metropolitan area: Seoul, Incheon, and Gyeonggi province.

^c^Nonmetropolitan area: cities and provinces other than the metropolitan area.

^d^K-MMSE-2: Korean-Mini-Mental State Examination, second edition.

^e^K-ADL: Korean-Activities of Daily Living.

^f^K-IADL: Korean-Instrumental Activities of Daily Living.

^g^HRQoL: health-related quality of life.

^h^PCS: physical component summary.

^i^MCS: mental component summary.

^j^CES-D: Center for Epidemiological Studies-Depression.

^k^MSPSS: Multidimensional Scale of Perceived Social Support.

Meanwhile, women were 55.1% less likely to be in the “high-level” digital literacy group than the “middle-level” group than men (OR 0.449, 95% CI 0.253-0.795). Regarding educational attainment, participants with less than a college education were 66.8% less likely to be categorized as “high-level” in digital literacy compared to those with below a college degree (OR 0.332, 95% CI 0.179-0.614). Those with less than a high school education were about 89.4% less likely (OR 0.106, 95% CI 0.050-0.223), and individuals with less than a middle school education were nearly 83.4% less likely (OR 0.166, 95% CI 0.068-0.405). Participants who had never worked were 76.6% less likely to be in the “high-level” digital literacy group compared to those currently engaged in income-generating activities (OR 0.234, 95% CI 0.061-0.902). Participants who did not regularly engage in physical exercise for more than 10 minutes were 42.2% less likely to be in the “high-level” digital literacy group than those who did (OR 0.578, 95% CI 0.397-0.843). Each one-month increment in the history of digital device use was associated with a higher likelihood of being in the “high-level” than “middle-level” digital literacy group (OR 1.009, 95% CI 1.006-1.013). In addition, each 1-point increase in perceived social support nearly doubled the likelihood of being in the “high-level” digital literacy group (OR 1.700, 95% CI 1.219-2.369).

## Discussion

### Principal Findings

This study identified distinct profiles of digital literacy among community-dwelling older adults using the EDLQ [26], an instrument developed for this demographic in accordance with the European Commission’s DigComp framework [11]. We categorized digital literacy into 3 distinct profiles: low level, middle level, and high level. Across all profiles, we observed a common pattern where digital literacy related to online information searching and social networking activities was relatively high. In contrast, skills in content creation and management, such as creating and editing documents, converting document formats, sharing content, and troubleshooting device issues, were notably lower. The low-level group exhibited particularly low skills in the security and safety domain, especially in their ability to block spam or delete search history. Meanwhile, the middle-level group displayed deficiencies in managing device security settings and seeking assistance for resolving technical issues within the security and safety domain.

### Comparison to Prior Work

Our results highlight 2 critical areas essential for supporting the digital literacy of older adults. The first relates to the ability to customize digital content according to individual needs. The second underscores the importance of literacy in digital security and safety. Concerns about security and safety, as noted in previous studies [46,47], can present a significant challenge for older adults when adopting new technology. These findings provide valuable insights that have not been adequately addressed in earlier research, which primarily concentrated on information search and online communication as benchmarks for evaluating digital literacy [13,48].

Notably, our findings highlight the presence of a digital divide among community-dwelling older adults in South Korea, emphasizing the need for tailored strategies to address this issue. Consistent with prior research [9,17,49,50], older adults who were female, of greater age, and had lower educational levels were more likely to exhibit low digital literacy. This suggests that despite high smartphone usage among older adults in countries like South Korea [51], a significant digital divide can still exist within this demographic. It further highlights the importance of considering sociodemographic factors when supporting digitally vulnerable populations [17-19]. For instance, in Singapore, a successful initiative known as “Project Wire Up” was implemented to ensure that older adults with lower socioeconomic status are not left behind in the digital society [52]. Skilled volunteers visited the homes of older adults to deliver a progressively challenging, tiered curriculum designed specifically for their needs. Consequently, the digital literacy scores of the participants in the intervention group improved significantly compared to those in the control group [52].

One approach to mitigating the digital divide among older adults is to increase the accessibility and use of digital devices. In our study, older adults with a longer history of using digital devices were more likely to achieve a middle-level of digital literacy rather than a low-level, and a high-level rather than a middle-level. Project Wire Up in Singapore [52] exemplifies this approach; a major component of the program was assisting older adults in acquiring smartphones and accessing the internet. In addition, the project focused on shifting traditional face-to-face social networking activities to digital platforms [52]. The recreational use of digital devices has been acknowledged as crucial for improving the digital literacy of older adults [23]. Furthermore, both the frequency and duration of internet use have shown a positive correlation with digital health literacy [53].

From a social connectedness perspective, the significance of social support and active participation in social activities is crucial for increasing the digital literacy of older adults. In our study, older adults who perceived greater social support were more likely to belong to the high-level digital literacy group rather than the middle level. Similarly, those engaged in in-person social activities tended to fall into the middle-level digital literacy group rather than the low-level group. A qualitative study investigating how older adults acquire digital literacy found that support from family, professionals, and peers significantly influenced not only the initiation but also the depth and frequency of digital device use [23]. Furthermore, a systematic review of the adoption of digital health technologies indicated that high social connectedness acts as a promoting factor across various cultural groups [54]. Although the exact mechanism of this association requires further exploration, social connectedness may impact digital literacy in older adults in 2 primary ways: first, by aiding them in learning and acquiring the skills to use digital devices [55], and second, by establishing a norm for digital device use [56].

In a similar context, our study showed that older adults currently engaged in economic activities were more likely to belong to the high-level digital literacy group compared to those who had never done so. Since economic activities often fall within the broader spectrum of social activities [57], it is plausible that the mechanism related to social connectedness also played a role here. In addition, previous research has shown that digital literacy in middle-aged and older adults positively affected their participation in managing personal finances [58]. Typically, most older adults may have primarily earned income through their careers until retirement. It is also conceivable that those more proficient in using digital devices and systems had better opportunities to engage in digital-based economic activities, such as using a mobile app for stock investments. However, since engagement in economic activities was measured with a single item, and the involvement of older adults in digital-based economic activities has not been extensively studied, our findings should be interpreted with caution and further research is needed.

Our findings also indicate that health-related factors are closely associated with digital literacy. Interestingly, our analysis revealed that older adults who are more dependent on IADL tend to fall into the middle-level digital literacy group rather than the low-level group. This contrasts with previous studies that focused on older adults with diabetes in South Korea [50,59]. This discrepancy suggests a potential new perspective on technology use among community-dwelling older adults. Since the early 2000s, there has been an increase in the use of smart home technology to assist individuals with diminished capabilities due to aging or disabilities [60-63]. These technologies, which incorporate sensors and robotics, can aid in supporting the IADL of older adults, such as meal preparation and bathing [60,61]. When introducing technology to older adults, the most critical factors to consider are the perceived benefits and ease of use [64,65]. Physical and cognitive aging can significantly hinder the adoption of new technologies by older adults [66]. As aging often requires more time to manage daily tasks like taking medication and undergoing physical therapy [67], older adults may view the adoption of new technologies as an additional burden that necessitates the reallocation of their time and energy [65].

In addition, in our sample, older adults who engaged in regular physical exercise were more likely to be categorized within the high-level digital literacy group. Similarly, a pilot study that used a smartphone app and activity-tracking device for physical training reported that participants with high technological proficiency were more receptive to modern technologies such as fitness trackers or smartphones compared to their less technologically adept counterparts [68]. Further research is required to explore the relationship between the health status or health behaviors of older adults and their use of digital technology.

### Limitations

This study has several limitations. First, it relies on cross-sectional data, which precludes making causal inferences about the relationships between variables. Further longitudinal research is necessary to determine the temporality of the variables identified. Second, although the study assessed a wide range of sociodemographic, health-related, and social variables, it is possible that some potential correlates of digital literacy were overlooked. In addition, significant variables measured by a single item, such as economic activity, may not allow for robust interpretation. Third, our assessment of digital literacy covered a broad spectrum of domains using a measurement that reflects the most recent definition of the concept. However, as digital literacy is an evolving field, future studies might find our assessment to be incomplete. Despite these limitations, this study effectively provides a current understanding and characterization of digital literacy among community-dwelling older adults in South Korea.

### Conclusions

Our findings reveal a significant disparity in digital literacy among older adults in a rapidly digitalizing country. Specifically, factors such as social connectedness and health status were closely associated with the levels of digital literacy observed within this population. Older adults with higher digital literacy may have more opportunities in the digital society. To prevent older adults, particularly those who are digitally vulnerable, from being marginalized in the digital society, we propose a tailored approach. This approach focuses on understanding the characteristics of these older adult groups and addressing modifiable factors. Strategies to enhance digital literacy among older adults could include improving access to digital devices and providing step-by-step instructions tailored to the needs and education levels of digitally vulnerable older adults. In addition, efforts are needed to promote social support and encourage participation in social activities.

## Abbreviations

CES-D: Center for Epidemiological Studies-Depression
DigComp: Digital Competence
EDLQ: Everyday Digital Literacy Questionnaire
K-ADL: Korean-Activities of Daily Living
K-IADL: Korean-Instrumental Activities of Daily Living
LPA: latent profile analysis
OR: odds ratio

## Data Availability

The datasets generated and analyzed during this study are available from the corresponding author on reasonable request.
